# Patients' and Clinicians' Perceptions of Clinician-Expressed Empathy in Advanced Cancer Consultations and Associations with Patient Outcomes

**DOI:** 10.1089/pmr.2020.0052

**Published:** 2020-06-11

**Authors:** Hinke Hoffstädt, Jacqueline Stouthard, Maartje C. Meijers, Janine Westendorp, Inge Henselmans, Peter Spreeuwenberg, Paul de Jong, Sandra van Dulmen, Liesbeth M. van Vliet

**Affiliations:** ^1^ Health, Medical and Neuropsychology Unit, Institute of Psychology, Leiden University, Leiden, The Netherlands.; ^2^ Department of Communication, Nivel (Netherlands Institute for Health Services Research), Utrecht, The Netherlands.; ^3^ Department of Medical Oncology, Netherlands Cancer Institute, Amsterdam, The Netherlands.; ^4^ Department of Medical Psychology, Amsterdam University Medical Centers, University of Amsterdam, Amsterdam, The Netherlands.; ^5^ Amsterdam Public Health Research Institute, Amsterdam, The Netherlands.; ^6^ Cancer Center Amsterdam, Amsterdam, The Netherlands.; ^7^ Department of Medical Oncology, St Antonius Hospital, Utrecht, The Netherlands.; ^8^ Department of Primary and Community Care, Radboud University Medical Center, Radboud Institute for Health Sciences, Nijmegen, The Netherlands.; ^9^ Faculty of Health and Social Sciences, University of South-Eastern Norway, Drammen, Norway.; ^10^ Leiden Institute for Brain and Cognition (LIBC), Leiden University, Leiden, The Netherlands.

**Keywords:** communication, empathy, palliative care, patient outcomes

## Abstract

***Background:*** Empathy is a cornerstone of effective communication. However, clinicians' and patients' perceptions of clinician-expressed empathy might differ. The independent perceptions of patients and clinicians on clinician-expressed empathy in advanced cancer consultations and the associations of these perceptions with patient outcomes are unknown.

***Objective:*** We assessed (1) patients' and clinicians' independent perceptions of clinician-(self-)expressed empathy in advanced cancer consultations and (2) the associations between these perceptions and affective patient outcomes.

***Methods:*** This observational study included data from 41 consultations in the advanced breast cancer setting. Postconsultation, patients' and clinicians' perceptions of clinician-expressed empathy were assessed, as well as patients' (1) pre–post anxiety, (2) post-anxiety, (3) emotional well-being, and (4) satisfaction. Multilevel regression analyses were run to draw conclusions.

***Results:*** Patients perceived higher levels of empathy than clinicians, without a significant relationship between the two (mean [*M*] = 85.47, standard deviation [SD] = 14.00 vs. *M* = 61.88, SD = 15.30, 0–100 scale; *β* = 0.14, *p* < 0.138, 95% confidence interval [CI] = −0.04 to 0.32). Higher patient-perceived empathy was associated with decreased anxiety [(1) *β* = −0.67, *p* = 0.039, 95% CI = −1.30 to −0.03; (2) *β* = −0.15, *p* = 0.042, 95% CI = −0.30 to −0.01], higher satisfaction (*β* = 0.05, *p* < 0.001, 95% CI = 0.03 to 0.08), and lower emotional distress (*β* = −0.32, *p* < 0.001, 95% CI = −0.48 to −0.16). There were no associations with clinicians' perceptions [(1) *β* = −0.34, *p* = 0.307, 95% CI = −1.00 to 0.31; (2) *β* = −0.02, *p* = 0.824, 95% CI = −0.17 to 0.14; (3) *β* < 0.01, *p* = 0.918, 95% CI = −0.03 to 0.02; (4) *β* = 0.08, *p* = 0.335, 95% CI = −0.08 to 0.25].

***Conclusions:*** Patients' and clinicians' empathy perceptions differed. In improving patient outcomes, the focus should be on patients' perceptions of clinician-expressed empathy. Future research could focus on ways to elicit patients' perceptions of empathy with the higher aim of improving patient outcomes.

## Introduction

Empathic clinician communication is one of the pathways through which patient outcomes are influenced (e.g., satisfaction and distress).^1–4^ Empathy expressions become especially important in cases of serious illness such as advanced cancer wherein stakes are particularly high.^5,6^ In these situations, empathy can decrease frequently occurring emotional distress such as anxiety and uncertainty, while increasing satisfaction.^7–10^

Although both patients and clinicians acknowledge the importance of clinician-expressed empathy,^11^ their perceptions of empathy use in clinical consultations might differ. For example, in a large study among hospitalized patients and their clinicians, both described empathy as very important and agreed it includes respectful communication and listening.^11^ Clinicians were, however, much more optimistic about whether empathy occurred, with 78% of clinicians versus 54% of patients believing that medical encounters entailed sufficient empathy.^11^

Similar patient–clinician disagreements on the use of empathy in consultations have been found in a wide variety of medical specialties, such as primary care, radiology, and surgical specialties.^12–14^ Given the importance patients place on receiving empathy,^15,16^ such disagreements might be undesirable.^17^ It is, however, unknown what the independent empathy perceptions are of patients and clinicians in the advanced cancer setting.

In addition, insight is needed on what the separate influences of patient-perceived clinician-expressed empathy (from now on referred to as patient-perceived empathy) and clinician self-perceived expressed empathy (from now on referred to as clinician-perceived empathy) are on patient outcomes. The association between patient-perceived empathy and improved affective outcomes such as satisfaction, emotional well-being, and anxiety has been demonstrated in a wide variety of studies, including those in oncology.^8,18–20^

However, research on the association between clinician-perceived empathy with patient outcomes has been limited. Some studies have shown an association between clinicians' awareness of their displayed empathy^21^ or their perception of the quality of the doctor–patient relationship^22^ with improved patient outcomes in diabetic care^21^ and primary care.^22^ Whether this also applies to the setting of advanced cancer is, however, unknown.

Against this background, this study aimed to determine (1) patients' and clinicians' independent perceptions of clinician-(self-)expressed empathy in advanced cancer consultations and (2) the associations between these perceptions and affective patient outcomes. Answering these questions can help shed light on the potential power of empathy in advanced cancer care from both patients' and clinicians' perspectives, which ultimately can be used to improve patient outcomes.

## Methods

### Design

This study is part of a larger observational research study in which clinician–patient consultations in advanced breast cancer care in two large hospitals in the Netherlands were audiorecorded. Both patients and clinicians were asked to complete questionnaires about the perceived communication (see our previous publication in which the methods are also described).^23^

### Ethical approval

The study was exempted from formal evaluation by the Medical Research Ethics Committee of the Dutch Cancer Institute. Both participating hospitals approved the study.

### Participants

Patients were eligible if they were women of >18 years of age with incurable breast cancer (determined by the clinical team), treated by one of the participating clinicians, had sufficient command of the Dutch language, and had sufficient cognitive ability to complete questionnaires. We focused solely on female patients since the majority of breast cancer patients are female and sex may influence (perceived) communication.^24,25^ Furthermore, patients were scheduled for either an initial consultation with regard to their incurable breast cancer or a consultation wherein evaluation (test) results would be discussed. Clinicians (oncologists) were included when they treated eligible patients and worked in one of the participating hospitals.

### Recruitment

Eligible patients were recruited by a contact person at the participating hospitals who enquired whether they would be interested in being approached by the research team investigating patient–clinician communication. When a patient approved, contact details of the interested patient were transferred to the research team (by telephone) after which a member of the research team would call the patient to provide more information regarding the study. There was no mention of the patients being in the advanced phase of their disease since not all patients may have been well aware of that. Patients were told they would be required to complete two questionnaires (one pre- and one postconsultation) and that their next scheduled consultation would be audiorecorded (this was necessary for the overall purpose of the larger study).

When preliminary consent was provided, the research team sent an information letter and consent form by (postal/electronic) mail to the patient, and the clinical team was informed of the preliminary consent. One of the researchers met the patient at the hospital just before their appointment to sign informed consent sheets. Patients were ensured that they were free to withdraw from the study at any time, that their data would remain anonymous, and were not expected to suffer from any harmful consequences due to the observational nature of the study. They were not compensated for their participation.

The participating clinicians were asked to participate by the research team or the coordinating investigator (one of the oncologists) of the hospital before the start of the study. They were also provided with an information letter and were asked to provide informed consent.

### Procedure

Preconsultation, patients completed a short questionnaire. Postconsultation, clinicians were asked to complete a questionnaire. When a clinician did not have sufficient time to complete the questionnaire at that moment, they returned the questionnaire by postal mail at a later time. The patients completed the second questionnaire at home with paper and pencil or through e-mail. A single reminder was sent to patients and clinicians if their questionnaire was not returned within two weeks after the consultation.

### Measures

#### Patient questionnaires

All questionnaires were developed with the input of patient representatives to ensure that completing the questionnaires would not be too burdensome.

##### Background demographics

Postconsultation, demographic characteristics of the patients such as age, marital status, ethnicity, level of education, occupation, and past/current oncological treatments were assessed.

##### Perceived empathy

Patient-perceived empathy was assessed postconsultation with a single 0–100 visual analogue scale (VAS) (ranging from “not at all” to “very much”) with the following question: “Can you indicate to what extent you felt that the clinician demonstrated empathy in the conversation that was audiorecorded.” The same VAS has been used in a previous study in a similar setting.^26^

##### Affective outcomes

###### Anxiety

First, preconsultation patients' anxiety about the upcoming consultation was assessed on a 0–100 VAS (ranging from “not at all” to “very much”) with the question, “Can you indicate how anxious you are at the moment?” The same VAS item was presented again postconsultation to assess the extent to which they felt anxious after the consultation. The same scale has been used before in a similar setting.^27^ Second, the state version of the State Trait Anxiety Inventory (STAI-s) was completed postconsultation.^28^ Patients responded to 10 questions on a four-point scale ranging from “not at all” to “very much,” leading to a total score ranging between 0 and 40. Examples of questions are “I feel nervous,” “I am worried,” and “I feel calm.”

###### Satisfaction

The extent to which patients were satisfied with the communication of the clinician during the consultation was assessed postconsultation with a single 1–10 item ranging from “not at all” to “very much” with the question, “Can you indicate the extent to which you are satisfied with regard to the communication by the clinician in the conversation that was audiorecorded?” An adapted version of this has been used before.^26^

###### Emotional well-being

Emotional well-being was assessed postconsultation with the five-item Emotion Thermometer Tool.^29^ The items of this tool are visualized as thermometers ranging from 0 (“not at all”) to 10 (“extremely much”), with a total score ranging between 0 and 50. The five different items assessed stress and tension, anxiety, depression, anger, and the need for help.

#### Clinician questionnaire

Postconsultation, clinicians were asked to complete a short questionnaire assessing perceptions of their own empathic communication and discussed information during the consultation.

##### Perceived empathy

The aforementioned 0–100 (“not at all” to “very much”) VAS item presented to patients was presented to clinicians postconsultation, enquiring after the extent to which they believed to have expressed empathy during the consultation with the question, “How much empathy did you express during the consultation?”

### Statistical analyses

First, the demographic characteristics of the sample of patients were described. Second, patient-perceived empathy and clinician-perceived empathy were described. Third, a multilevel linear regression analysis was run with patient-perceived empathy as the independent variable and clinician-perceived empathy as the dependent variable to assess whether patients' and clinicians' perceptions were significantly related. Fourth, to facilitate interpretation, the analysis was run again with standardized scores for both the patient- and clinician-perceived empathy variables, so that the coefficient of the analysis could be interpreted as a correlation between clinician and patient perception.

Next, four multilevel linear regressions were run to assess the associations of patient-perceived empathy and clinician-perceived empathy with patient outcomes. Each regression had patient-perceived empathy and clinician-perceived empathy as their independent variables. Each multiple linear regression had an affective patient outcome as dependent variable: (1) pre–post anxiety, (2) postanxiety, (3) patient satisfaction, and (4) emotional well-being. All analyses controlled for clustering of results to the different clinicians and hospitals by adding clinician as a level. Since solely two hospitals participated in the study, hospital was not suitable to add to the model as a level (Intraclass Correlation Coefficient <0.01), but was added to the model as a factor variable instead.

Analyses were run with STATA 14.0. All tests were two sided and considered significant when *p* < 0.05.

## Results

### Participants

As described in detail previously,^23^ of the 84 approached patients, 39 were not included (no oral consent (*n* = 19), not fulfilling inclusion criteria (*n* = 4), not reachable (*n* = 2), logistical problems (*n* = 10), withdrawing of consent (*n* = 2), and audiorecording failed (*n* = 2). Of the 45 included patients, 41 completed all questionnaires. All approached 12 oncologists agreed to participation.^23^ Background demographics are summarized in Table 1 (which is reprinted from our previously published article using this dataset).^23^

**Table 1.** Background Characteristics Participants

	Total (*n* = 41^a^)
	*M* (SD)
Age (years)	57.18 (12.20)
	Range 31–84
	*n* (%)
Marital status	
Married	27 (66)
Single (including divorced, widowed)	14 (34
Highest Education^b^	
Low	—
Intermediate-1	9 (22)
Intermediate-2	18 (44)
High	14 (34)
Occupation	
Paid job	10 (24)
Disabled/sick leave	14 (34)
Housewife	4 (10)
Retired	13 (32)
Ethnicity	
Dutch	35 (86)
Western immigrants^c^	5 (12)
Non-Western immigrant	1 (2)
Treatments currently receiving^d^	
Chemotherapy	18 (44)
Radiotherapy	2 (5)
Hormone therapy	16 (39)
Immunotherapy	9 (22)
Operation	—
Targeted therapy	4 (9)
Symptom-oriented treatment	10 (24)
Tumor-oriented treatment possible, but refrained from	—
Tumor-oriented treatment impossible	1 (2)

This table is reused.^23^

^a^ Out of the 45 participating women, 41 completed all questionnaires, data of the remaining 4 could not be retrieved.

^b^ Low = primary education or less (<12 years of age); intermediate-1 = lower secondary (>12 years of age); intermediate-2 = upper secondary (>12 years of age); high = tertiary (university/college) (>18 years of age).

^c^ Someone is considered a Western immigrant when he/she or at least one of the parents was born in Europe (excluding Turkey), North America, Oceania, Indonesia, or Japan. All immigrants from different backgrounds are considered non-Western immigrants.

^d^ Participants can receive several treatments, so this does not add up to 100%.

*M*, mean; SD, standard deviation.

### Independent perceptions of patients and clinicians on clinician-expressed empathy

Patient-perceived empathy was higher than clinician-perceived empathy (mean [*M*] = 85.47, standard deviation [SD] = 14.00, range: 44.50–100.00 vs. *M* = 61.88, SD = 15.30, range: 20.20–84.80). A multilevel regression analysis showed no significant relationship between clinician- and patient-perceived empathy (*β* = 0.14, *p* < 0.138, 95% confidence interval = −0.04 to 0.32). The multilevel regression analysis run again with standardized scores of empathy scores of patients and oncologists enabled us to interpret the beta coefficient as a correlation that was very low (*r* = 0.12).

### Associations between patient- and clinician-perceived empathy and patient outcomes

As illustrated in Table 2, with four multilevel regression analyses, we assessed the associations of patient-perceived empathy and clinician-perceived empathy with four affective outcomes. Pre–post difference in anxiety was associated with patient-perceived empathy (*p* = 0.039), but not with clinician-perceived empathy (*p* = 0.307). Postconsultation anxiety was also associated with patient-perceived empathy (*p* = 0.042), but not with clinician-perceived empathy (*p* = 0.824). Satisfaction too was associated with patient-perceived empathy (*p* < 0.001), but not with clinician-perceived empathy (*p* = 0.918). Emotional well-being was also associated with patient-perceived empathy (*p* < 0.001), but not with clinician-perceived empathy (*p* = 0.335).

**Table 2.** Associations between Perceptions and Affective Patient Outcomes

	Patients' perceptions			Clinicians' perceptions		
	β	95% CI	*p*	β	95% CI	*p*
Anxiety pre–post	−0.67^*^	−1.30 to −0.03	0.039	−0.34	−1.00 to 0.31	0.307
Anxiety post	−0.15^*^	−0.30 to −0.01	0.042	−0.02	−0.17 to 0.14	0.824
Patient satisfaction	0.05^*^	0.03 to 0.08	<0.001	<0.01	−0.03 to 0.02	0.918
Emotional well-being	−0.32^*^	−0.48 to −0.16	<0.001	0.08	−0.08 to 0.25	0.335

^*^ *p* < 0.05.

CI, confidence interval.

## Discussion

The aim of this study was to determine (1) patients' and clinicians' independent perceptions of clinician-(self-)expressed empathy in advanced cancer consultations and (2) the associations between these perceptions and affective patient outcomes. We found that patients perceived significantly higher levels of clinician-expressed empathy than clinicians. Patient-perceived empathy was not related to clinician-perceived empathy. Patient-perceived empathy was associated with all four affective outcome measures, influencing them positively, whereas clinician-perceived empathy was not associated with any outcome.

Our finding that patients perceived more clinician-expressed empathy than clinicians themselves perceived contradicts most previous research,^11,14,30^ but not all.^31^ Several clinician and patient factors specific to the advanced cancer setting might explain this, as previous studies were mostly conducted within primary care.

Focusing on clinician factors, clinicians regularly experience stress and insecurity when dealing with incurably ill patients due to discomfort around the topic of death and the act of dismissing hope.^32–35^ We could speculate that this discomfort might instill the belief in clinicians that they are less empathic, or might lead to a more critical evaluation of their expressed empathy (and rating yourself highly might be uncomfortable) than primary care settings, which induces less discomfort due to the less seriously ill population. In addition, self-assessment of clinicians has been known to not always be accurate.^36^ Alternatively, given the increasing attention on clinicians'—empathic—communication skills,^37^ we could speculate that oncologists become increasingly aware of their behaviors. This might also help explain our contradictory findings to the previous—earlier—research.^11,14,30,37^

Moving to patient factors, an explanation could be that patients' evaluation of clinician-expressed empathy is influenced by the longstanding clinician–patient relationship in serious illness. Patients' sense of loyalty, trust, and satisfaction increases when they are more familiar with their doctor.^38–42^ Furthermore, patients with (advanced) cancer have reported to experience an instant sense of trust in their oncologist due to their vulnerable situation.^43^ Lastly, when patients are generally satisfied, the likelihood to dismiss potential communicative missteps of the doctor increases (e.g., the “halo effect”).^44^ In sum, in the advanced cancer setting, clinicians might be more self-critical, whereas their patients might be more lenient when assessing their clinicians' empathic behavior, compared with primary care settings.

The second part of the study was dedicated to the associations of patient-perceived clinician-expressed empathy and clinicians' self-perceived empathy with affective patient outcomes. Our finding that patient-perceived empathy is associated with decrease in anxiety and distress and increase in satisfaction is in line with previous findings showing similar effects on psychological well-being and patient satisfaction.^8–10,19^ However, the lack of association of clinicians' self-perceived empathy with affective patient outcomes is in contrast with some previous literature demonstrating that clinicians' perceptions can play a role in patients' emotional well-being, satisfaction, and quality of life.^21,22,45^

Previous studies were, however, mostly conducted with patients suffering from nonlife-threatening illness and may, therefore, have yielded different results. It is arguable that in the setting of advanced cancer, the importance of patient-perceived empathy grows due to the high stakes involved^6^ and the great amounts of distress and uncertainty experienced^46^ and, therefore, may exceed the importance of clinicians' perceptions.

### Practical implications

When trying to improve patient outcomes through optimal communication, our study showed that the focus should be on patients' perceptions of empathy as opposed to clinician self-perceived empathy. Clinicians should thus be aware of how their empathic communication skills are perceived by their patients, which does not always seem to be the case.^47^ This lack of awareness as to how they are perceived might make it difficult for clinicians to adjust their empathic behavior where necessary. It is not uncommon for doctors to make assumptions regarding patients' perceptions and needs during medical encounters.^48,49^ By asking patients about their own perceptions, clinicians' empathic behavior might be optimized and patient outcomes ultimately be improved.

As different patients have different needs, this enquiry might need to be done for each patient so that communication can be tailored.^50,51^

### Future research

The most prominent question that comes to light based on the results of this study is what clinicians could do to have an accurate perception of how their empathic communication skills are perceived by their patients. Question prompt lists (QPL) to facilitate question-asking behavior of patients and patient-reported outcome measures (PROMs) to facilitate better insight into patients' well-being and needs have turned out to be effective in improving clinician–patient communication in oncology, leading to favorable patient outcomes.^52–55^

QPLs and PROMs designed to encourage patients to express their perceptions, needs, and preferences regarding their doctors' empathic communication might increase clinicians' awareness as to how they are perceived and how they could optimize their use of empathy. Future research could focus on the possibility of designing a tool to help elicit emotional needs of patients with focus on clinicians' empathy use in advanced cancer consultations and investigate its potential to improve patient-perceived empathy and ultimately patient outcomes.

Alternatively, clinicians could directly ask patients to offer feedback during consultations regarding their empathic communication. Doctors receiving direct feedback from patients through the means of a short feedback form with open-ended questions and opportunity for direct follow-up have shown to be effective in improving communication, as well as patients' and physicians' satisfaction.^56^ Future research could further explore this possibility of a teach-back method with clinicians asking for direct feedback during consultations regarding their empathic communication in the setting of advanced cancer and its effect on patient outcomes.

### Limitations

This study has several limitations. First, the sample size was small; results in a larger well-powered sample might differ.

Second, participants were all female, most were highly educated, and the majority of patients were treated in a cancer-specialized hospital.

Third, a bias may have been present in our sample, limiting generalizability of results, since it is possible that patients who were uncomfortable or unsatisfied with their clinician were less inclined to participate. Also the participating oncology teams might be more interested in communication and have better communication skills than nonparticipating teams.

Fourth, since empathy was not defined in the questionnaires, responses were based on each patients' subjective concept of what empathy entails. Since each patient may have a different idea of what empathy entails, no conclusions can be drawn on which specific behaviors of the clinicians were appreciated by the patients and improved patient outcomes.

Lastly, the formulation of the empathy question and the questionnaire completion moment (most oncologists: immediately postconsultation, patients: at home—although exact completion moment was not assessed) for oncologists and patients differed, possibly influencing results.

## Conclusion

This study demonstrates the power of patient-perceived empathy to improve patient outcomes.^6,9,10^ In advanced cancer settings, patients seem to perceive more clinician-expressed empathy than clinicians themselves, and these perceptions can decrease their anxiety while increasing satisfaction and emotional well-being. So, patient-perceived clinician-expressed empathy should be the core focus when trying to improve medical communication and optimizing patients' well-being at a time this is most crucial.

## Abbreviations Used

CI: confidence interval
*M*: mean
NIAS-KNAW: Netherlands Institute for Advanced Study in the Humanities and Social Sciences
PROM: patient-reported outcome measure
QPL: question prompt lists
SD: standard deviation
STAI-s: State Trait Anxiety Inventory
VAS: visual analogue scale
